# Osteoblastoma in the Proximal Phalanx of the Hand: A Case Report

**DOI:** 10.7759/cureus.48409

**Published:** 2023-11-06

**Authors:** Adrian Joseph C Tablante, John Hubert C Pua, Ervan Thierry A Tan

**Affiliations:** 1 Orthopaedic Surgery, Jose R. Reyes Memorial Medical Center, Manila, PHL

**Keywords:** pediatric, tumor, proximal phalanx, hand, osteoblastoma, case report

## Abstract

Osteoblastoma is a benign bone tumor that can spread aggressively and is commonly found in the spine and long bones. When present in other areas of the body, it can be difficult to diagnose. While this tumor is rarely found in the hand, in reported cases, it typically presents with pain. Treatment is usually curettage and marginal excision. We report a rare case of osteoblastoma in the fifth proximal phalanx of the left hand in a 14-year-old right-handed female, presenting as a painless, progressively growing mass with associated flexion contracture over a seven-month period, with no history of trauma. An excision biopsy with curettage was performed, and histopathologic examination confirmed the diagnosis of osteoblastoma. This is a rare case of osteoblastoma of the proximal phalanx presenting as a painless mass in the finger with a progressive flexion contracture. Histopathologic examination is important in diagnosing osteoblastoma to determine the appropriate treatment and surgery. Post-operatively, close monitoring is important due to the high recurrence rates in these tumors.

## Introduction

Osteoblastoma is a rare, aggressive benign tumor that accounts for approximately 0.5%-2% of all primary bone tumors and 3% of all benign bone tumors with a predilection for the axial skeleton, followed by the craniofacial bones [1, 2]. This tumor can occur at any age but predominantly affects the younger population, with a peak incidence in the second and third decades with a male-to-female ratio of approximately 2:1 [1]. Osteoblastomas rarely present in the hand, with an incidence of less than 5% in the phalanges [2, 3].

Osteoblastomas are usually identified as a mass larger than 1.5 cm in diameter, which causes pain that remains constant throughout the day and cannot be relieved by salicylates [3]. On the other hand, osteoid osteoma is characterized by a nidus smaller than 1.5 cm and pain that can be alleviated by salicylates [1]. The primary treatment for osteoblastomas is curettage or marginal resection with or without bone grafting, with additional radiotherapy if necessary [1, 4, 5]. For recurrent tumors, surgical en bloc resection may be necessary; however, the recurrence rate may be as high as 25% even after surgical resection [1].

The aim of this paper is to illustrate the presentation, diagnosis, and treatment of a rare case of osteoblastoma located at the proximal phalanx of the hand. The presentation of this osteoblastoma was atypical owing to its location and clinical presentation, making the diagnosis difficult and emphasizing the need to consider this condition in bony tumors of the hand.

## Case presentation

**Figure 1:**
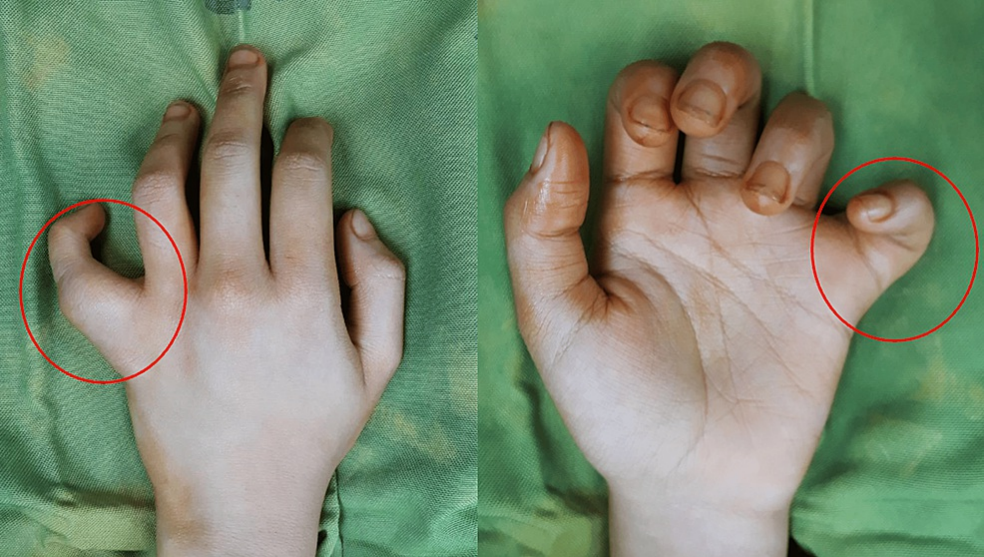
Dorsal and volar views of the left hand show a mass in the little finger.

A 14-year-old right-handed female student was referred by a general practitioner for a seven-month history of a left little finger mass. She noted a painless lump on the proximal phalanx of her left little finger and an associated difficulty in extending the involved finger. There was no history of antecedent trauma. Clinical examination revealed a 2.5 x 2.0 cm, hard, non-tender mass on the radial side of the little finger, with a 50° contracture angle of the proximal interphalangeal joint (Figure 1).

**Figure 2:**
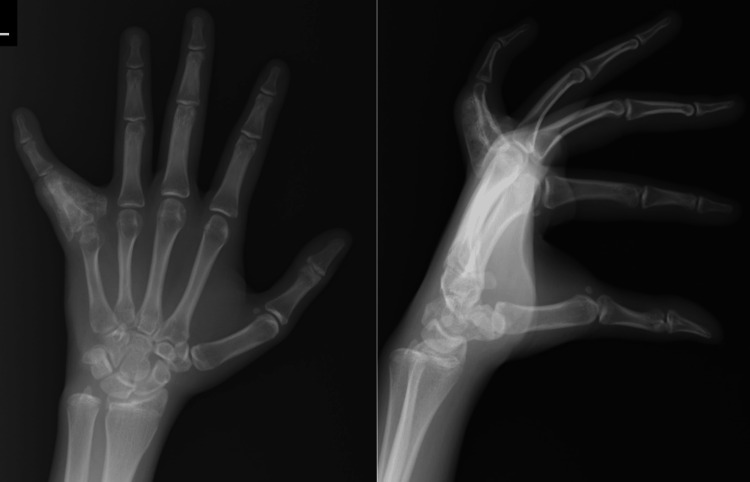
Anteroposterior and lateral view radiographs of the left hand show a large periosteal reaction on the proximal phalanx of the left little finger.

The X-ray films revealed a periosteal reaction with associated lytic lesions on the proximal phalanx of the left little finger, described as a florid reactive periostitis (Figure 2).

**Figure 3:**
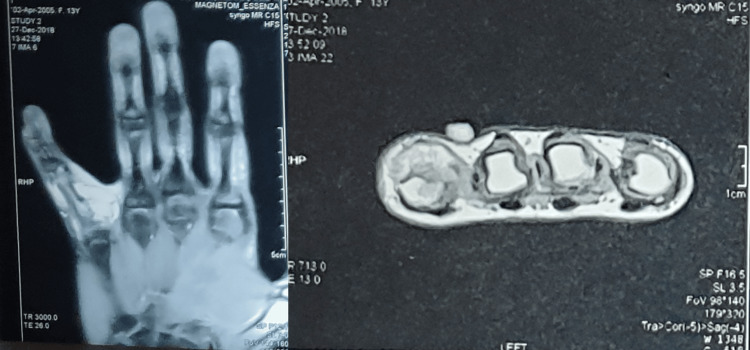
Coronal and axial views of the MRI scan of the left hand show a lesion surrounding the proximal phalanx of the little finger.

A plain MRI was done, showing a lesion of the dorsal aspect of the proximal phalanx of the affected finger sized 1.4 x 2.0 x 2.1 cm with surrounding subcutaneous edema and mild erosive changes of the affected phalanx (Figure 3).

**Figure 4:**
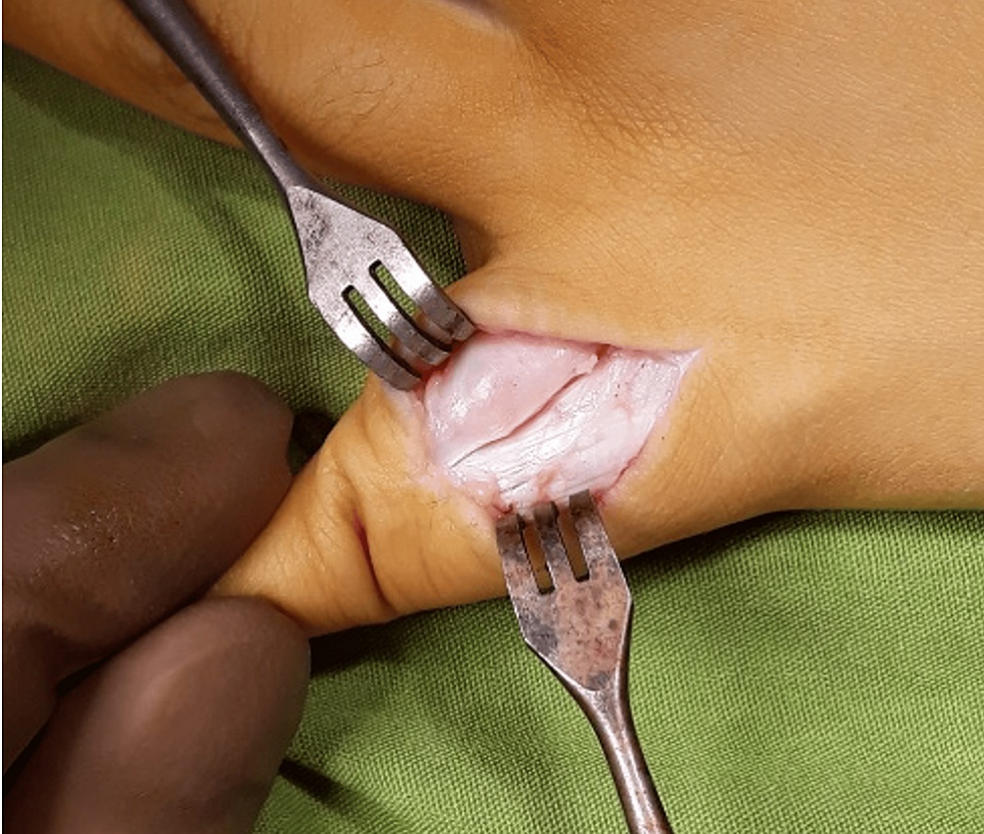
Dorsal longitudinal incision with exposure of the lesion of the proximal phalanx of the left little finger

The initial diagnosis was an enchondroma. The patient underwent a marginal excision biopsy with curettage through a dorsal longitudinal approach after an intraoperative frozen section confirmed a benign process (Figure 4).

**Figure 5:**
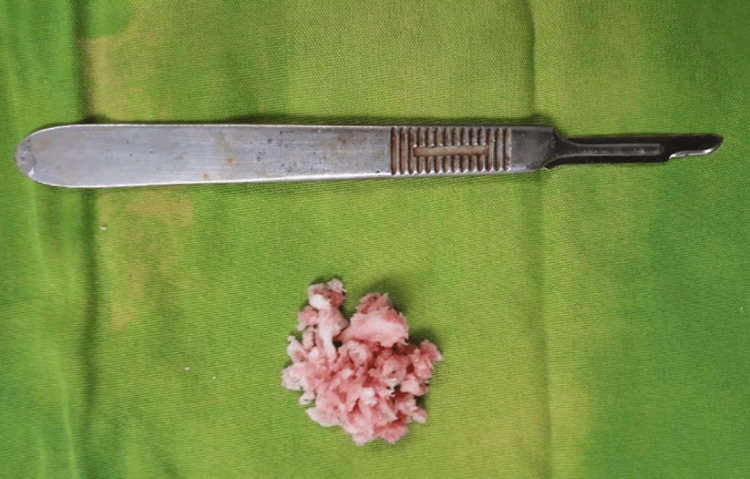
Excised tumor of the proximal phalanx next to a scalpel handle as a reference

The bony lesions were noted to have an intact surrounding shell and were excised (Figure 5).

**Figure 6:**
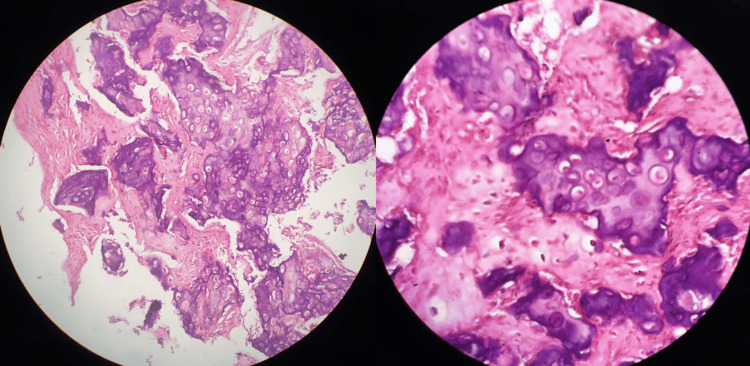
Histologic sample of osteoblastoma showing anastomosing trabeculae of osteoid and woven bone with a single layer of benign activated osteoblasts and numerous osteoclasts

The collected specimen was sent for histopathologic examination, which showed anastomosing trabeculae of osteoid and woven bone rimmed by a single layer of benign activated osteoblasts and numerous osteoclasts (Figure 6).

**Figure 7:**
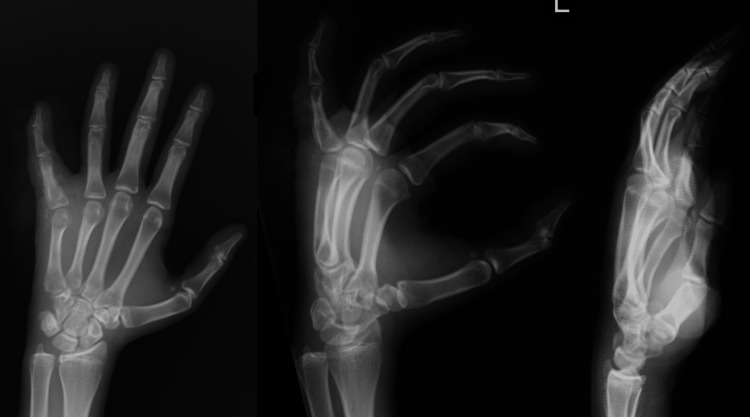
Post-operative anteroposterior, oblique, and lateral view radiographs of the left hand with resolution of the periosteal reaction of the proximal phalanx of the little finger

The final diagnosis was an osteoblastoma of the proximal phalanx of the left little finger. Post-operative X-ray films revealed interval resolution of the periosteal reaction around the proximal phalanx (Figure 7).

**Figure 8:**
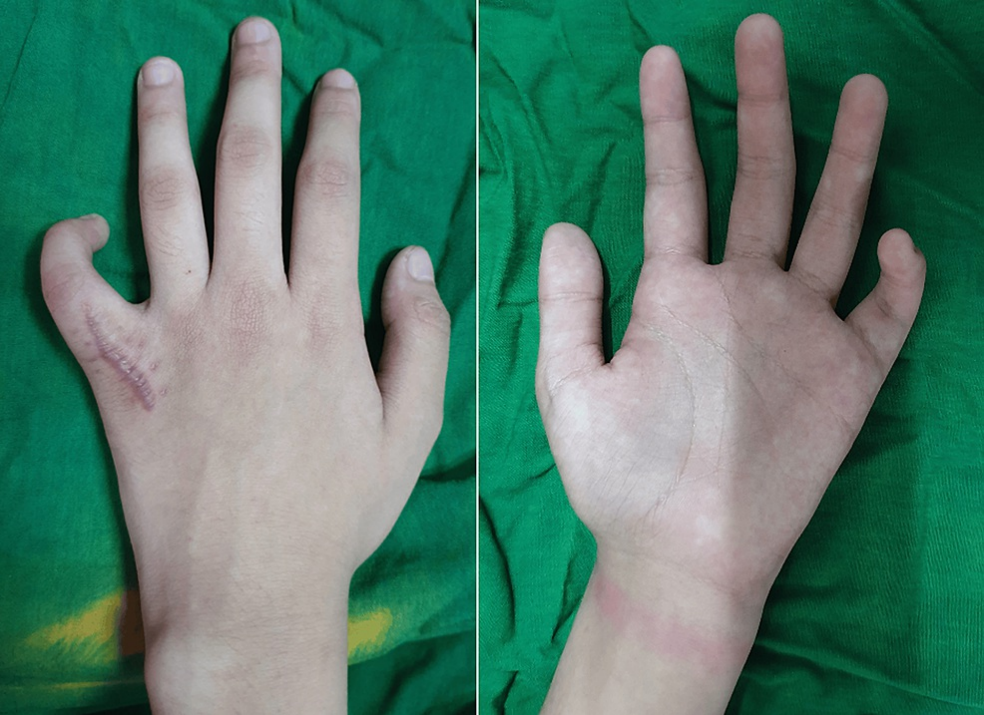
Dorsal and volar views of the left hand eight weeks after the operation show a flexion contracture of the little finger and a surgical scar on the dorsal aspect.

The finger was kept in a simple, sterile gauze dressing until wound healing occurred two weeks post-operatively. There was a noted inability to actively extend the involved finger, still with a contracture angle of 50° of the proximal interphalangeal joint (Figure 8).

Eight weeks post-operatively, the finger was placed on an extension splint, and the patient was started on a rehabilitation program. The patient was advised on the need for long-term follow-up due to the aggressiveness of the lesion and the possibility of recurrence. Unfortunately, due to the COVID-19 pandemic, the patient was lost to follow-up and could not be contacted for a consultation.

## Discussion

Osteoblastoma is a benign, rare, and aggressive tumor initially described as a “giant osteoid osteoma” due to its clinical and histological similarities to that of an osteoid osteoma [6]. In 1956, it was differentiated from an osteoid osteoma and arbitrarily designated as an osteoblastoma if the nidus is more than 1.5 cm in diameter [7, 8]. Radiographic features of benign osteoblastomas are similar to those of osteoid osteomas, which are described as a round or oval, well-demarcated metaphyseal lytic defect surrounded by a zone of reactive sclerosis [1, 9].

Osteoblastomas that exceed 4 cm in size and show prominent periosteal new bone formation may be mistaken for osteosarcomas [1, 10]. Histologically, osteosarcomas often have atypical features, including foci of lace-like osteoid deposition, high cellularity, and scattered mitotic figures [1, 11]. Tumors exhibiting these features may be misinterpreted as malignant and should be evaluated further [1, 10]. In rare instances, there is also a possibility of malignant transformation [12, 13, 14].

Computed tomography (CT), bone scans, and MRIs may be utilized to aid in diagnosis. A CT may provide information about the size and extent of the lesion in the cortical bone to aid in preoperative evaluation [15]. Bone scans and MRIs are nonspecific. However, an MRI can more accurately reveal intra-osseous and soft-tissue extensions [9, 16].

These tumors may expand the bone contour with a markedly thinned cortex and cause bone destruction, with or without periosteal new bone formation [1]. Radiographic presentation suggestive of malignancy, such as cortical destruction and extra-osseous soft tissue expansion, was present in 12% of cases [3, 9, 17]. The gross appearance of osteoblastomas is typically a well-demarcated, hemorrhagic nidus with markedly expanded bone contours [1]. Microscopically, the nidus tissue is described to consist of an interlacing network of bone trabeculae and a prominence of osteoblasts and multinucleated osteoclast-like giant cells [1].

Primary treatment of osteoblastomas is typically simple curettage or marginal resection with or without bone grafting [4]. Surgical en bloc resection should be limited to recurrent tumors and aggressive forms of the disease [5]. However, the recurrence rate may reach as high as 25% even after resection [1, 2]. Recurrences rarely occur after two years of initial surgery; therefore, close, constant follow-up of patients is required for the first two years [1, 18].

A differential diagnosis for this case was an enchondroma, which is the most common primary bone tumor found in the hand, particularly in the proximal phalanx [19]. It typically presents non-specifically with pain, swelling, deformity, or a pathologic fracture in 40%-60% of patients [19]. Radiographically, enchondromas usually do not present with a periosteal reaction; hence, we were considering other possibilities, such as an atypical presentation or other malignant bone tumors, such as chondrosarcoma [19]. However, histopathologic examination confirmed an unusual presentation of osteoblastoma, given that this tumor rarely occurs in the hand. An osteoid osteoma was also initially considered due to its histological similarity to osteoblastoma but ruled out due to the large size of the lesion [6]. Osteoblastomas are most commonly found in the axial skeleton, presenting in 40% of cases, and are exceptionally rare in the hand, with less than 5% presenting in the phalanges [1, 2, 9]. There are only a few case reports that describe an osteoblastoma of the phalanx. One case report describes an osteoblastoma on the distal phalanx of the ring finger, which was successfully treated with curettage and polymethylmethacrylate filling [15]. Another case report similar to our current study describes a benign osteoblastoma of the proximal phalanx of the left little finger, successfully treated by surgical resection and curettage [16]. Furthermore, the clinical observation of a painless mass on the finger was atypical, as these tumors are typically linked to a history of slowly progressive dull aching pain, as seen in previous case reports of osteoblastomas of the hand [3, 5, 15, 16, 20].

In our patient, a dorsal approach was utilized since the tumor presented on the dorsal aspect of the affected finger. Marginal excision of the tumor was done with significant removal of the tumor mass and some soft tissue extension, which most likely caused the inability to actively extend the affected finger. Our patient was started on a rehabilitation program and advised on long-term follow-up due to the high likelihood of recurrence and possible progression of this tumor.

## Conclusions

Osteoblastomas in the hand and wrist are uncommon and usually cause localized pain, which is contradictory to the case of our patient. To the best of our knowledge, there are only a few case reports that describe osteoblastomas in the proximal phalanx of the hand. The clinical and radiographic similarities with other bone tumors highlight the importance of accurately confirming the diagnosis to limit recurrence. By describing our pediatric patient's case of osteoblastoma with an unusual presentation, we hope to assist in the diagnosis and treatment of future cases involving bony tumors of the hand.
